# Novel Site-Specific Fatty Chain-Modified GLP-1 Receptor Agonist with Potent Antidiabetic Effects

**DOI:** 10.3390/molecules24040779

**Published:** 2019-02-21

**Authors:** Xia Zhong, Zhu Chen, Qiong Chen, Wei Zhao, Zhi Chen

**Affiliations:** 1 College of Life Science and Technology, Jinan University, Guangzhou 510000, China; 2 Reyoung Biopharmaceuticals Co., Ltd, Suzhou 215000, China; Zchen@163.com (Z.C.); qchen0862@yeah.net (Q.C.); zwei1008@163.com (W.Z.); 3 East China University of Science and Technology, Shanghai 200000, China; Paulchan121@outlook.com

**Keywords:** Glucagon-like peptide-1 receptor, extracellular domain, Exendin-4, fatty chain, antidiabetic effects

## Abstract

Glucagon-like peptide-1 receptor (GLP-1R) agonists have emerged as treatment options for type 2 diabetes mellitus (T2DM). Here, we designed a high-throughput GLP-1R extracellular domain (ECD)-based system that enabled the screening of high-potency receptor-biased GLP-1R agonists demonstrating new pharmacological virtues. Firstly, six 12-mer peptides (termed PEP01–06), screened from a large phage displayed peptide library were fused to the N-terminus of Exendin-4 (29–39) to generate PEP07–12. By the use of four lysine-altered PEP07 (PEP13–16) as the starting point, a series of fatty chain conjugates (PEP17–20) were synthesized and evaluated by in vitro GLP-1R-based cell assays. In addition, the acute and long-term in vivo effects on diet-induced obesity (DIO) mice were further evaluated. All four conjugates showed good receptor activation efficacy, and PEP20 was selected to undergo further assessment. Preclinical experiments in DIO mice demonstrated that PEP20 had significant insulinotropic activities and glucose-lowering abilities. Moreover, a prolonged antidiabetic effect of PEP20 was also observed by the hypoglycemic test in DIO mice. Furthermore, long-term treatment with PEP20 achieved beneficial effects on the food intake, weight gain, hemoglobin A1C (HbA1C) lowering activity, and glucose tolerance compared with the control and was similar to the Liraglutide. In conclusion, PEP20, a GLP-1R ECD-biased agonist, may provide a novel therapeutic approach to T2DM.

## 1. Introduction

Type 2 diabetes mellitus (T2DM) is a complex metabolic disorder characterized by hyperglycemia arising from insufficient insulin secretion and insulin resistance [1,2]. Glucagon-like peptide-1 (GLP-1) is a 30-amino-acid peptide hormone secreted from gut endocrine L-cells in response to nutrient ingestion, and plays an important role in glucose homeostasis and nutrient metabolism [3,4,5]. GLP-1 lowers postprandial glucose excursion by potentiating glucose stimulated insulin secretion from pancreatic B cells in a glucose-dependent mode. In addition, GLP-1 outperforms other oral hypoglycemic agents in promoting gastric emptying, weight loss and increasing insulin sensitivity in peripheral tissues. Hence, GLP-1-based therapies represent a strategy for the treatment of T2DM [6,7,8]. For example, Exendin-4, a 53% homology with native GLP-1, is a clinically-approved full agonist with a stronger affinity for GLP-1R and longer in vivo half-life [9,10,11,12]. 

GLP-1, or Exendin-4, exerts its action through the GLP-1R, which is expressed in a number of organs, including the pancreas, the central nervous system and the peripheral tissues. GLP-1R, is defined by seven transmembrane helices, an extracellular N terminus, and an intracellular C terminus, which is ligand binding via the extracellular and intracellular face, respectively, belongs to family B1 of the seven-transmembrane G protein-coupled receptors [13,14,15,16,17]. Both GLP-1 and Exenind-4 interact with the GLP-1R by binding multiple extracellular contact points to induce receptor signaling. Glucagon-like peptide-1 receptor extracellular domain (ECD), as an “addinity trap” to sense the GLP-1 or its analogs outside of the cell, leads to an elevation of cyclic adenosine monophosphate (cAMP), modulates intracellular calcium concentration and induces b-arrestin recruitment, which plays an important role in insulin synthesis and release of insulin in a glucose-dependent mode [18,19,20]. X-ray crystal structures of Exendin-4 and GLP-1 bound to the ECD confirm the “affinity trap” hypothesis showing the C-terminal α-helical region of GLP-1 or Exendin-4 is positioned within a binding cleft of the N-terminal ECD [21,22]. Despite the weaker affinity of GLP-1 for isolated ECD (IC_50_~100 nM) than the full-length GLP-1R (IC_50_~1 nM), GLP-1R ECD still could be used as a useful high throughput in vitro assessment methods for screening the GLP-1R agonists [23,24].

In this work, we developed a GLP-1R ECD-based system to screen the large phage displayed 12-mer peptide library with the aim of identifying receptor-biased GLP-1R agonists. Then six peptides were fused to the Exendin-4 (29–39) to generate the hybrid ones with higher affinity for GLP-1R. We identified the selected one such hybrid peptide and then covalent coupled to fatty chain, termed PEP20. Moreover, both the in vitro and in vivo characteristics of PEP20 were investigated to identify its antidiabetic and obesity properties. 

## 2. Results 

### 2.1. Discovery of Novel Glucagon-Like Peptide-1 Receptor Agonists

**Figure 1:**
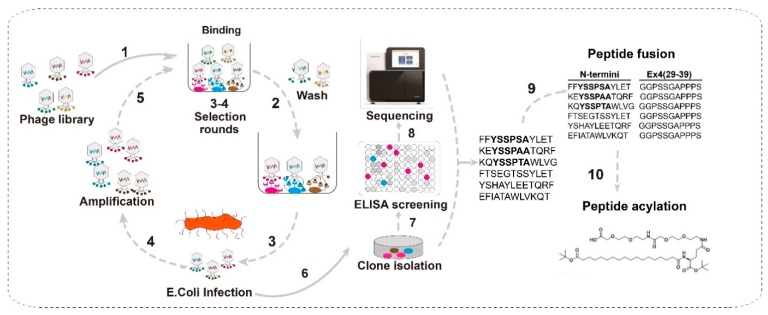
Selection of Glucagon-like peptide-1 receptor agonists from peptide libraries.

To discover the potential peptides with high affinity for GLP-1R a commercial peptide library, designed based on M13 phage and containing linear 12 random peptide sequences, was subjected for biopanning the GLP-1R ECD-binding domain (Figure 1). Six peptides, with high affinity for GLP-1R ECD, were selected from peptide libraries using a phage display technique and defined as PEP01–PEP06. 

The sequence GGPSSGAPPPS of Exendin-4, as the key C-terminal region which could enhance its affinity for the receptor, interacts with the N terminus ECD of GLP-1R, facilitating the receptor activation [20,25]. On basis of these Exendin-4 structure–activity insights, the selected short GLP-1R agonists (PEP01–06) peptide library consisting of random 12 amino acids fused to Exendin-4(29–39) was generated, which accounted for the increased receptor activation activity of Exendin-4 compared with GLP-1. As a result, six hybrid peptides termed PEP07–PEP12 were constructed after the fusion of two sequences. All of these hybrid peptides were identified to exhibit higher affinity for GLP-1R ECD after fusion (Table 1). 

SPR measurements showed a significant higher binding affinity of PEP07 for GLP-1R ECD than either peptide, yielding a K_d_ of 24 nM. Considering the predictable short half-life in vivo, these candidate molecules still should be further modified to enhance bioavailability. Therefore, PEP07 was selected as the lead peptides for further acylation.

### 2.2. Synthesis and Characterization of the Fatty Chain-Modified Hybrid Glucagon-Like Peptide-1 Receptor Agonists

**Figure 2:**
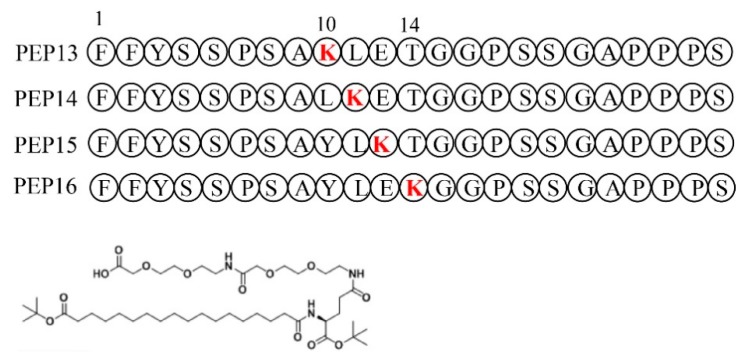
Structure of fatty chain-modified Glucagon-like peptide-1 receptor agonist conjugates.

Lysine scanning mutagenesis was performed to determine a suitable acylation site. The four consecutive amino acids (YLET) of PEP07 were individually replaced by lysine to generate four peptides (Figure 2, named PEP13–16). The associating affinity of Lys-PEP07 peptides for GLP-1R ECD are shown in Table 2. Although lysine mutagenesis on a few sites led to decreased affinity (e.g., PEP15), lysine replacement at some sites did not affect the receptor binding potency, such as PEP14 and PEP16. Particularly, PEP16, with highest associating affinity for GLP-1R ECD (25 nM), was selected for further site specific acylation. 

As shown in Table 3, the selected lysine-altered peptides (PEP13–16) were reacted with fatty chains to give four fatty chain-modified conjugates (PEP17–20) which were purified by preparative reverse phase high-performance liquid chromatography (RP-HPLC) and further identified by liquid chromatrography tandem mass spectrometry (LC–MS).

### 2.3. In Vitro Activity of Hybrid Glucagon-Like Peptide-1 Receptor Agonist

As shown in Table 4, slight reduction in the activation potency was observed due to the fatty chain modifications. Notably, the modified conjugates (PEP17 and PEP18), close to a consensus motif of “YSSPXA”, which could enhance its affinity for the receptor, were less potent to the modified conjugates, such as PEP20, which were relatively far away from the probable active domain. Since the EC_50_ of PEP20 in both human or mouse GLP-1R activation was similar to the reference peptide Exendin-4, and better than the native GLP-1 or Liraglutide, we selected this molecule for the further efficacy evaluation in vivo.

### 2.4. Glucoregulatory and Insulin Secretion Assay

**Figure 3:**
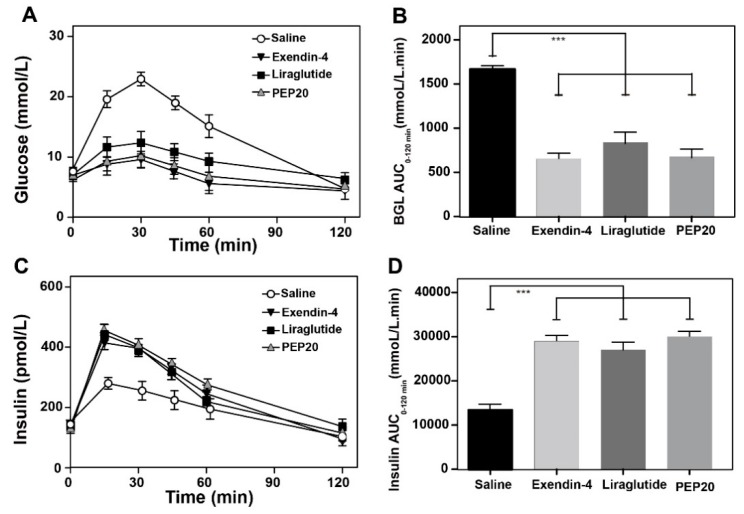
Insulin secretion and glucose tolerance tests in diabetic mice. (**A**) Glucose-lowering and (**B**) insulinotropic activities of Exendin-4, Liraglutide, and PEP20 (25 nmol/kg) in diet-induced obesity (DIO) mice. (**C**) Area under the curve (AUC)_glucose_ and (**D**) AUC_insulin_ after oral glucose administration. Results are presented as means ± SD (*n* = 6 each group). *** *p* < 0.001.

Oral glucose tolerance test was performed at 1 h after diet-induced obesity (DIO) mice received 25 nmol/kg PEP20, Exendine-4 or Liraglutide to evaluate the glucose-lowering and insulinotropic effects (Figure 3A,B). Half an hour after a glucose challenge, the blood glucose level (BGL) in the vehicle treated group rapidly increased to 23.6 ± 3.1 mmol/L while in the PEP20-, Exendin-4-, and Liraglutide-treated mice, dramatically reduced to 10.1, 9.8, and 12.2 mmol/L, respectively. Moreover, a glucose-lowering percentage of 60.2% was showed in PEP20 treated group, while the Exendine-4 or Liraglutide group showed a reduction of 66.1% or 46.1%, respectively, compared with the vehicle control group. 

Similar time courses for plasma insulin concentrations in PEP20, Liraglutide or Exendin-4 treated group were observed and all plasma insulin concentrations from 15 to 60 min were significantly greater than those of the control group. In particular, PEP20 showed similar and comparable insulin secretion promoting ability than Exendin-4 and Liraglutide, respectively (Figure 3C,D).

### 2.5. Hypoglycemic Duration Test

**Figure 4:**
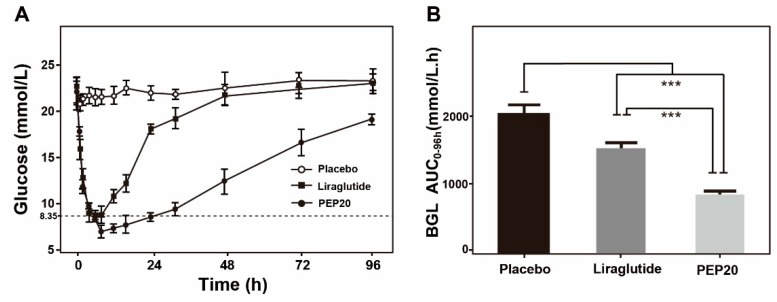
Glucose-lowering and stabilizing effects of Liraglutide and PEP20 were determined by hypoglycemic duration test in DIO mice. (**A**) Antihyperglycemic efficacies of Liraglutide and PEP20 in DIO mice pretreated with each sample (25 nmol/kg) for 96 h. (**B**) Hypoglycemic effects of control, Liraglutide and PEP20 based on AUC_0–96 h_. Results are presented as means ± SD (*n* = 6 each group). *** *p* < 0.001.

The antihyperglycemic efficacy of PEP20 was investigated in male DIO mice. The BGLs of PEP20 group decreased rapidly to the normoglycemic state (<15 mmol/L) in 1 h after the administration of 25 nmol/kg. The lowest BGLs in the PEP20 group were similar to the Liraglutide group (Figure 4A). Moreover, the glucose-lowering effect of PEP20 was maintained up to at least 96 h, but only about 24 h in Liraglutide treated group. Furthermore, PEP20 significantly lowered the glucose level of 63.8% for 0–96 h compared with the vehicle group (Figure 4B), while it was only 23.2% after the Liraglutide treatment. These results revealed PEP20 has better antihyperglycemic efficacy and hypoglycemic duration performance than the commercial Liraglutide at the same dose.

### 2.6. Chronic In Vivo Studies 

**Figure 5:**
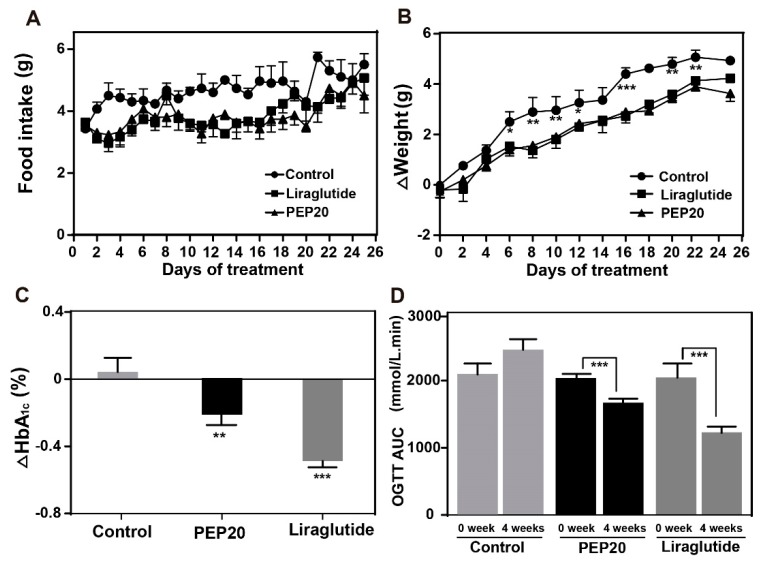
The effects of chronically administered PEP208 on DIO mice. (**A**) Food intake. (**B**) Body weight gain. (**C**) HbA1C. (**D**) Chronic OGTT AUC. Results are presented as means ± SD (*n* = 6 each group). * *p* < 0.05, ** *p* < 0.02, *** *p* < 0.001.

To further examine the therapeutic potential of PEP20, chronic in vivo studies were performed by a daily subcutaneous (s.c.) injection of PEP20 (25 nmol/kg) in DIO mice for 4 weeks. As is indicated in Figure 5A,B, the body weight gains and food intake amounts were effectively suppressed in PEP20 treated mice, to a comparable extent to Liraglutide treated ones. Moreover, as is showed in Figure 5C, both PEP20 and Liraglutide treatment resulted in a reduction in HbA1c values (*p* < 0.001, *n* = 6) than that in control group. 

OGTT tests were performed to evaluate whether a long-term treatment of PEP20 helped to improve the glucose metabolism in DIO mice. The results, graphed in Figure 5D, revealed that there is no significant difference between the area under the curve (AUC) of saline treated group before or after 4 weeks administration. However, we found that the AUC of PEP20 and Liraglutide treated group at 4 weeks decreased 22.1% or 38.2% compared with week 0. These results showed that PEP20 exerted a significant improvement in the glucose metabolism of DIO mice which was similar to Liraglutide, a commercialized GLP-1R agonist.

## 3. Discussion

G protein-coupled receptor (GPCR) peptide ligands have always been noted for their selectivity, potency, and rapid optimization [26]. Although typically peptides can be developed by chemical synthesis, they have obvious disadvantages, such as a low-throughput process that intrinsically lacks diversity. To address this, we have developed affinity-based screening technologies from a random peptide library with a storage capacity of more than one billion peptides. In the proof-of concept experiments presented here, we show how this methodology allows one to detect and identify novel GLP-1R ligands with unique pharmacology.

GLP-1, an endogenous polypeptide hormone released in response to food intake, potentiate insulin secretion from pancreatic cells through binding and activation of its receptor, and GLP-1R agonists have emerged as treatment options for type 2 diabetes mellitus. [6,18,27]. Although the treatments with GLP-1R agonists demonstrate the significant acute or long-term antidiabetic activities [28,29], there were a great many problems such as unavoidable side effects and the homogenization because almost all the related drugs were developed according to the only two native GLP-1R agonists (GLP-1 and Exendin-4) [30,31,32]. Recently, a novel concept of ‘biasing seven-transmembrane receptors’, for the developing drugs have been getting increased attention for its potential greater therapeutic effects while possibly avoiding unwanted side effects [33]. The GLP-1R consists of a predicted signal sequence, the ECD of approximately 120 residues, seven membrane-spanning α-helices connected by three extracellular and three intracellular loops (the TM domain), and a C-terminal intracellular domain [20]. It is well established that the N-terminal ECD of incretin receptors is important for ligand binding and ligand specificity, whereas the transmembrane domain is involved in receptor activation [15]. Moreover, the GLP-1R ECD, with ligand binding function, can be expressed in both eukaryotic and prokaryotic systems, such as *Escherichia coli* and mammalian cells [34,35]. 

Here, a GLP-1R ECD-based system to screen the large phage displayed 12-mer peptide library with the aim of identifying receptor-biased GLP-1R agonists was successfully established (Figure 1). As a result, six 12-mer peptides (PEP01–06) were successfully screened from a large phage displayed peptide library (Table 1). Interestingly, three of the peptides contained a motif of YSSPXA (where X is S, A, or T), which could enhance its affinity for the receptor. Then the fusion of six selected peptides to the Exendin-4(29–39) was confirmed to significantly enhance their affinity for GLP-1R (Table 1). Considering the predictable short half-life in vivo, we selected the PEP07, with the highest affinity for GLP-1R ECD, as the leader peptide for the further lysine mutagenesis and side chain modification of fatty acid to determine whether different conjugation sites affected the in vitro activity (Figure 2 and Table 2). A series of fatty chain conjugates (PEP17-20) were synthesized and evaluated by in vitro GLP-1R-based cell assays and results PEP20 showed similar effects on the activation of both human and mouse GLP-1R compared to Exendin-4 (Table 4). 

It was not only that PEP20 showed similar and comparable promoting insulin secretion ability than Exendin-4 and Liraglutide, respectively, correspond with the in vitro results (Figure 3). The long-acting glucose-lowering effects of PEP20 were further confirmed by the hypoglycemic duration in DIO mice. As we expect, the glucose-lowering effect of PEP20 was maintained up to at least 96 h, which was significant longer than Liraglutide, a commercial GLP-1R agonist (Figure 4). Chronic in vivo studies on DIO mice showed that the body weight gains and food intake amounts were effectively suppressed in PEP20 treated group, to a comparable extent to Liraglutide treated ones (Figure 5A,B). Moreover, as is showed in Figure 5C, both PEP20 and Liraglutide treatment resulted in a reduction in HbA1c values (*p* < 0.001, *n* = 6) than that in control group. At week 0, glucose tolerance in both control, Liraglutide and PEP20 treatment group is similar, but at 4 weeks, effect of PEP20 on improvement of impaired glucose tolerance in DIO mice was predominantly compared with vehicle control group (Figure 5D).

Moreover, what deserves more investigation is that PEP20 showed significant better hypoglycemic duration than Liraglutide while both the improvement on the HbA1c lowering and glucose tolerance of DIO mice were a little weaker than Liraglutide. We hypothesized that might be possibly because that newly designed PEP20 owns a lower sequence homology than Liraglutide compared with native GLP-1 or the coupled fatty chain which lead to a weaker long-term effects on DIO mice. Considering that PEP20, in spite of its prolonged glucose lowering ability, may not the best choice to be a candidate to bring to clinics, we did not further investigate the pharmacokinetic properties of it. Somewhere along the line, we will further to evaluate PEP20 in some other animal model, such as db/db mice or optimize the sequence combination (e.g., PEP07 and GLP-1 (9–37)) and fatty chain of PEP20.

In summary, GLP-1R-based binding screening of phage display peptide libraries enabled the discovery of high-potency GLP-1R agonists. A series of preclinical studies demonstrates that the fusion of selected GLP-1R agonist and Exendin-4 (29–39) showed a significant enhancement in the affinity for GLP-1R ECD. Then the further side fatty chain modified hybrid peptide PEP20 showed well preserved in vitro receptors activation activity and long-acting in vivo incretin-based antidiabetics. Moreover, chronic in vivo studies on DIO mice also showed that PEP20 is a promising agent deserving further investigation to treat obesity patients with diabetes. Not only that, this strategy may also be applicable to the discovery and design of other GPCR agonists.

## 4. Materials and Methods

### 4.1. Materials and Animals

Ph.D.-12 phage library (New England Biolabs, Beverly, MA, USA) was subjected to biopanning for GLP-1R agonist peptides. All the other peptides, with or without side chain modification, were obtained from Bankpeptide Biological Technology Co. (Hefei, China) used a solid phase method. INS-1 cells were obtained from China Infrastructure of Cell Line Resources (Beijing, China). Male 6- to 8-week-old DIO mice were purchased from GenePharma Co., Ltd. (Suzhou, China). Our animal experiments were approved by the Animal Ethics Committee with the approval codes NO. 2017R-131, NO. 2018R-194 and NO. 2018R-263.

### 4.2. Phage Display

The Ph.D.-12 phage library containing linear 12 random peptide sequences with the complexity of ~109, was applied to discover novel GLP-1R agonist. The GLP-1R ECD, dissolved in 0.01 M PBS (pH 7.0), was coated on 96-well plates. Four rounds of screening peptides with high affinity for GLP-1 ECD were conducted according to the instruction of phage library kit. The selected phage was propagated with *E. coli* ER 2738 strain. The accurate sequences were obtained using the universal primer 96 gIII (5´-CCCTCATAGTTAGCGTAACG –3´) for the subsequent DNA sequencing.

### 4.3. Characterization of Glucagon-Like Peptide-1 Receptor Agonists

Molecular weights of GLP-1R agonists were detected using an Autoflex III smartbeam-matrix-assisted laser desorption ionization time-of-flight mass spectrometer (MALDITOF/MS, Bruker, Karlsruhe, Germany).

### 4.4. Surface Plasmon Resonance Measurements

Surface Plasmon Resonance (SPR) measurements were performed with Biacore 8K (GE Health, Boston, MA, USA) for evaluating the associating affinity peptides for GLP1R ECD. Experimental details were carried out according to the Biacore 8K user’s manual.

### 4.5. In Vitro Activity of Glucagon-Like Peptide-1 Receptor Agonists

INS-1 cells, after two days maintenance, were added with 200 μL RPMI 1640 medium free of glucose and then the medium was replaced by a fresh RPMI 1640 added with 2.2 or 16.8 mM glucose and different concentrations of GLP-1R agonists for 2 h. The secreted insulin was detected using a commercial ELISA kit (Merck, Darmstadt, Germany).

### 4.6. Oral Glucose Tolerance Tests in Fasted Diet-Induced Obesity Mice

The male DIO mice received a single subcutaneous injection of saline, Exendin-4, Liraglutide and PEP20 (25 nmol/kg). Half hour after single dose injection of different samples, the mice were administrated with glucose (2.0 g/kg), and then the blood glucose and insulin levels were detected before and at 15, 30, 45, 60 and 120 min by the ELISA kit (Merck).

### 4.7. Hypoglycemic Efficacy in Not Fasted Diet-Induced Obesity Mice 

Male DIO mice, with the same average body weight, blood glucose levels and same water and food supply, received a single subcutaneous injection of saline, PEP20 (25 nmol/kg) or Liraglutide (25 nmol/kg). Blood samples were collected from tail veins at different time-points (0, 1, 2, 4, 6, 8, 12, 18, 24, 36, 48, 72, and 96 h). BGLs were detected using a one-touch blood glucose meter. Glucose-lowering duration under 8.35 mmol/L BGL was also checked

### 4.8. Chronic In Vivo Studies 

The DIO mice were subcutaneously injected with saline, Liraglutide, PEP20 (25 nmol/kg) once daily for 4 weeks. Body weight, food intake, and water consumption were measured daily. DIO mice were subjected to OGTT at week 0 and at week 4 to evaluate the advance of diabetic statuses. HbA1c were measured after the treatment using an AU680 automatic biochemical analyzer (Beckman Coulter, Fullerton, Germany).

### 4.9. Data Analysis

Data were analyzed on GraphPad Prism 5 (GraphPad Software, Inc., La Jolla, CA, USA). All measured variables were presented by mean ± SD. Differences in all parameters were tested via one-way ANOVA; *p*-value < 0.05 indicates statistical significance.

## Figures and Tables

**Table 1:** The binding affinity of peptide 01–12 for Glucagon-like peptide-1 receptor extracellular domain.

Peptide	Sequence	K_d_ (μM)	Peptide	Sequence	K_d_ (μM)
01	FF**YSSPSA**YLET	0.74	07	FF**YSSPSA**YLET GGPSSGAPPPS	0.024
02	KE**YSSPAA**TQRF	1.05	08	KE**YSSPAA**TQRF GGPSSGAPPPS	0.151
03	KQ**YSSPTA**WLVG	1.75	09	KQ**YSSPTA**WLV GGPSSGAPPPS	0.253
04	FTSEGTSSYLET	3.99	10	FTSEGTSSYLET GGPSSGAPPPS	2.998
05	YSHAYLEETQRF	2.21	11	YSHAYLEETQRF GGPSSGAPPPS	1.252
06	EFIATAWLVKQT	2.59	12	EFIATAWLVKQT GGPSSGAPPPS	1.092

**Table 2:** The binding affinity of PEP13–16 for Glucagon-like peptide-1 receptor extracellular domain.

Peptide	Sequence	K_d_ (μM) (Unconjugated)	K_d_ (μM) (Conjugated)
PEP13	FFYSS PSA*K*L ETGGP SSGAP PPS	0.098	0.158
PEP14	FFYSS PSAY*K* ETGGP SSGAP PPS	0.053	0.062
PEP15	FFYSS PSAYL *K*TGGP SSGAP PPS	0.033	0.091
PEP16	FFYSS PSAYL E*K*GGP SSGAP PPS	0.023	0.025

**Table 3:** Characterization of the fatty chain-modified Glucagon-like peptide-1 receptor agonists.

Peptide	Retention (min)	Molecular Mass		Conjugated-Peptide	Retention (min)	Molecular Mass	
		Calculated	Found			Calculated	Found
PEP13	5.62	[M + 3H]^+^ 756.2	756.3	PEP17	6.65	[M + 3H]^+^ 989.0	989.2
		[M + 4H]^+^ 567.1	567.2			[M + 4H]^+^ 741.8	741.9
PEP14	5.46	[M + 3H]^+^ 772.8	772.9	PEP18	6.39	[M + 3H]^+^ 1005.6	1005.8
		[M + 4H]^+^ 579.6	579.5			[M + 4H]^+^ 754.2	754.5
PEP15	5.72	[M + 3H]^+^ 767.5	767.4	PEP19	6.81	[M + 4H]^+^ 1000.3	1000.1
		[M + 4H]^+^ 575.6	575.5			[M + 3H]^+^ 750.3	750.1
PEP16	5.23	[M + 3H]^+^ 776.8	776.6	PEP20	5.97	[M + 3H]^+^ 1009.4	1009.1
		[M + 4H]^+^ 582.6	582.5			[M + 4H]^+^ 757.1	756.9

**Table 4:** In vitro pharmacological characterization of the GLP-1 Receptor peptide.

Peptide	Human GLP-1R		Mouse GLP-1R	
	EC_50_ (nM)	*E*max (%)	EC_50_ (nM)	*E*max (%)
Exendin-4	0.052 ± 0.012	100 ± 2.5	0.076 ± 0.008	100 ± 1.1
Liraglutide	0.282 ± 0.041	81 ± 5.2	0.185 ± 0.039	89 ± 2.1
GLP-1	0.552 ± 0.89	76 ± 7.2	0.322 ± 0.199	81 ± 4.6
PEP13	0.064 ± 0.022	96 ± 4.1	0.051 ± 0.009	110 ± 9.4
PEP14	0.051 ± 0.008	105 ± 9.2	0.034 ± 0.002	150 ± 10.9
PEP15	0.451 ± 0.121	78 ± 9.1	0.457 ± 0.085	73 ± 6.4
PEP16	0.111 ± 0.054	92 ± 7.1	0.101 ± 0.012	93 ± 9.3
PEP17 (Conjugated)	0.412 ± 0.019	79 ± 6.4	0.678 ± 0.012	68 ± 5.9
PEP18 (Conjugated)	0.391 ± 0.081	78 ± 6.4	0.351 ± 0.044	78 ± 3.5
PEP19 (Conjugated)	0.152 ± 0.092	89 ± 6.2	0.212 ± 0.102	83 ± 3.2
PEP20 (Conjugated)	0.062 ± 0.011	98 ± 8.1	0.051 ± 0.003	99 ± 7.2
